# To Our Readers

**Published:** 1887-12-10

**Authors:** 


### To our Readers.

We have reason to think that it has been industriously circulated, for a purpose we need not dwell upon, that The Hospital is merely the organ of The Hospitals Association, and that it has no title to claim to be an independent organ of public opinion. It may be well, therefore, that we should state that the Association is as independent of this journal, and The Hospital of the Association, as the latter is independent of any other publication or paper that may be named. The Hospital is of course the organ of hospitals and asylums and nursing institutions, and their governors and officials, in a very real and practical sense, and so our wish and desire is to promote the work of The Hospitals Association in every possible way. This we have always done, and shall ever endeavour to do, but merely as an act of grace and from a genuine desire to help one of the most useful associations of our day and generation. The policy of The Hospital was clearly stated in the first number, published October 2nd, 1886. We have not, nor do we intend to depart from the comprehensive programme then fully set forth, and the alteration in the sub-title when we increased the size of The Hospital in October last was merely to emphasise briefly but clearly the ground covered by this journal.

Our mission is then to soothe the sufferers, to aid the injured, to comfort the dying, and to help the sick, and, as we said at the outset, "the wider such influences are extended the better for the nation and the people at large. Between the parish or congregation, the family or household, and even the individual, and the Hospital and its workers, there is that touch of nature, that golden chord of human sympathy, which makes the whole world kin." Every parish, congregation, family, household, and individual must encounter the dark days of human weakness, of which disease is "the infallible magnet, as all must one day realise in their own persons, or in the persons of those who are nearest and dearest to them. This being so it will be the mission of The Hospital to aid all to provide against this day of trial, to face it happily, and to fight disease with the best weapons available," and especially "by extending the area of public interest and knowledge in the work of those who labour amongst sickness and suffering." This we have steadily done from first to last, and it is not one of the least of the laurels The Hospital has won during its career that it should have stepped into the breach when others refused to continue to expend the necessary money, and by the publication of special supplements should have aided the Council of the Metropolitan Hospital Sunday Fund to successfully continue the new departure initiated at the instance of the late Dr. Wakley. The result has been, as all must know, that in this year of Jubilee, despite other attractions and special expenditure of all kinds, the people of London have given through the Hospital Sunday Fund at the call of the clergy and ministers of religion of all denominations £40,607, the largest sum ever raised for the hospitals through the Metropolitan Fund. Here is eloquent testimony to the value of co-operation amongst all classes, churches, and creeds on behalf of the hospitals. Such efforts, when supported by the press, must increasingly multiply funds by extending the general interest in these institutions. An earnest desire to provide all the hospitals with adequate funds to enable them to minister to all who applied to them for succour—combined with a loving regard for our noble-hearted friend, the late Dr. Wakley—were the sole motives which led the conductors of this journal to help to maintain and extend the Hospitals Week; and so no notice of this action of The Hospital at a critical period in the history of the Hospital Sunday Fund appears in the report of the Council which will be laid before the constituents at the annual general meeting, to be held in the old council chamber of the Guildhall, on Monday next, at three p.m., the Right Hon. P. De Keyser, Lord Mayor, president and treasurer, in the chair. For this reason it was the more gratifying to receive a letter on Wednesday from one of the leaders of religious thought in the metropolis, who is also a member of the Council of the Hospital Sunday Fund, and personally unknown to the writer, in which the following statement is made: "The Hospital is a varied, cheery, and every way excellent paper." The clergy and all who work amongst the sick, being earnest workers themselves, are of all people the most grateful for honest and hearty co-operation in the good work of helping the sick; and we thank them cordially for the many marks of favour and approval they have extended to this journal.

The special field which we now occupy had never been filled until The Hospital was published. From the encouragement and support so liberally extended to us, it is no idle boast to say we shall continue to cultivate it, with the co-operation of an ever-increasing number of parishes, congregations, families, practitioners, and individuals who have come forward each week in larger numbers to help us in this great humanising effort to do a noble work. For weeks past we have never printed less than 10,000 copies of The Hospital, and last week requirements were so great that 20,000 copies were called for. This result is due to the kindness and enthusiasm of our many readers and friends, both in this country, in the Colonies, and in America. To them be the greater honour and all the praise. For ourselves, we are content to know that we have no cause to trim our sails or to look askance at any principles or aims with which we commenced this work, in what we believe to be the best interests of the poor, the hospitals, the profession, and the public at large; nor shall we ever be tempted to depart from the platform of straightforward, honest, outspoken, and fair dealing between every interest, institution and individual, be they whom they may, with which we commenced our labour of love.

